# Physical and Pharmacokinetic Characterizations of *trans*-Resveratrol (*t*-Rev) Encapsulated with Self-Assembling Lecithin-based Mixed Polymeric Micelles (*sa*LMPMs)

**DOI:** 10.1038/s41598-017-11320-y

**Published:** 2017-09-06

**Authors:** Tzu-Pin Li, Wan-Ping Wong, Ling-Chun Chen, Chia-Yu Su, Lih-Geeng Chen, Der-Zen Liu, Hsiu-O Ho, Ming-Thau Sheu

**Affiliations:** 1 School of Pharmacy, College of Pharmacy, Taipei Medical University, Taipei, Taiwan, ROC; 2 Department of Biotechnology and Pharmaceutical Technology, Yuanpei University of Medical Technology, Hsinchu, Taiwan, ROC; 3 Department of Microbiology, Immunology, and Biopharmaceutics, National Chiayi University, Chiayi, Taiwan, ROC; 4 Graduate Institute of Biomedical Materials and Engineering, Taipei Medical University, Taipei, Taiwan, ROC; 5 Clinical Research Center and Traditional Herbal Medicine Research Center, Taipei Medical University Hospital, Taipei, Taiwan, ROC

## Abstract

This study involved physical and pharmacokinetic characterizations of trans-resveratrol (*t*-Rev)-loaded *sa*LMPMs which attempted to improve *t*-Rev’s pharmacokinetic profiles and bioavailability resolving hurdles limiting its potential health benefits. The optimal formulation consisted of *t*-Rev, lecithin, and Pluronic^®^ P123 at 5:2:20 (*t*-Rev-loaded PP123 *sa*LMPMs) provided mean particle size <200 nm, encapsulation efficiency >90%, and drug loading >15%. Compared to *t*-Rev solubilized with HP-β-CD, *t*-Rev-loaded PP123 *sa*LMPMs enhanced *t*-Rev’s stability in PBS at RT, 4 °C, and 37 °C and in FBS at 37 °C, and retarded the *in vitro* release. Intravenous administration of *t*-Rev-loaded PP123 *sa*LMPMs was able to enhance 40% absolute bioavailability and a greater portion of *t*-Rev was found to preferably dis*t*ribute into peripheral compartment potentially establishing a therapeutic level at the targeted site. With oral administration, *t*-Rev-loaded LMPMs increases 2.17-fold absolute bioavailability and furnished a 3-h period of time in which the plasma concentration maintained above the desirable concentration for chemoprevention and accomplished a higher value of the dose-normalized area under the curve for potentially establishing an effective level at the target site. Therefore, intravenous and oral pharmacokinetic characteristics of *t*-Rev encapsulated with PP123 *sa*LMPMs indicate that *t*-Rev can be translated into a clinically useful therapeutic agent.

## Introduction

Over the past few decades, *trans*-resveratrol (3,4′,5-trihydroxy-*trans*-stilbene; *t*-Rev) was reported to possess antioxidant, anti-inflammatory, anticarcinogenic, antidiabetic, antiaging, cardioprotective, and neuroprotective properties, which can be relevant in chronic diseases and longevity in humans^1^. A narrative review of the anticancer and chemopreventive molecular mechanisms of *t*-REV summarized that by simultaneously acting on diverse mechanisms, *t*-Rev was emphasized as a promising, multi-target, anticancer agent, relevant for both cancer prevention and treatment^2^. Considering all the beneficial effects of *t*-Rev on human health, drug supplements containing *t*-Rev in traditional dosage forms, including tablets, capsules, and micronized powders for oral delivery, have been developed to translate into clinical applications in the past several decades^1, 3–10^. However, the single biggest problem in *t*-Rev’s clinical translation appears to be its rapid metabolism leading to limited *in vivo* bioavailability as revealed by Singh *et al*.^11^.

*In vitro* data previously showed that for the chemopreventive effects to be effective, it was necessary for free *t*-Rev to achieve a minimum plasma concentration of 5 μM^3^. However, following oral administration in humans, 75% of *t*-Rev may be absorbed possibly via transepithelial diffusion, but oral bioavailability of free *t*-Rev was found to be low (<1%), which was confirmed to be a result of rapid and extensive metabolism in the intestines and liver^5, 12^. Nevertheless, despite low levels of free *t*-Rev being detected in the plasma after oral administration of free *t-*Rev, numerous beneficial effects of *t*-Rev were still reported^13^. Since rapid metabolism seems to be a limiting factor in translating *t*-Rev’s promising chemopreventive and chemotherapeutic effects to humans, researchers began to focus on different means of enhancing the bioavailability of *t*-Rev, including i) co-administration with *t*-Rev metabolism inhibitors in order to prolong its presence *in vivo*, ii) the use of *t*-Rev analogs that possess better bioavailability, iii) investigations into the activity potential of *t*-Rev metabolites, and iv) drug delivery system design focused on nanotechnologies^14^. Among these, designing drug delivery systems capable of enhancing *t*-Rev’s bioavailability is particularly promising.

Different strategies for formulating nanocarrier systems with the ability to enhance *t*-Rev’s aqueous solubility and oral bioavailability over traditional forms were reported, including dripping pills^15^, self-nanoemulsifying drug delivery systems^11, 16, 17^, solid lipid nanoparticles^18, 19^, polymeric nanoparticles^20–22^, and liposomes^23^. Among those *in vivo* studies with oral administration of *t*-Rev at 25~2000 mg per dose in humans or 10~50 mg per kg in animals, *t*-Rev levels in plasma mostly remained at concentrations below or considerably below 5 µM (~1150 ng/mL), which is required to elicit pharmacologic effects relevant to chemoprevention.

**Figure 1:**
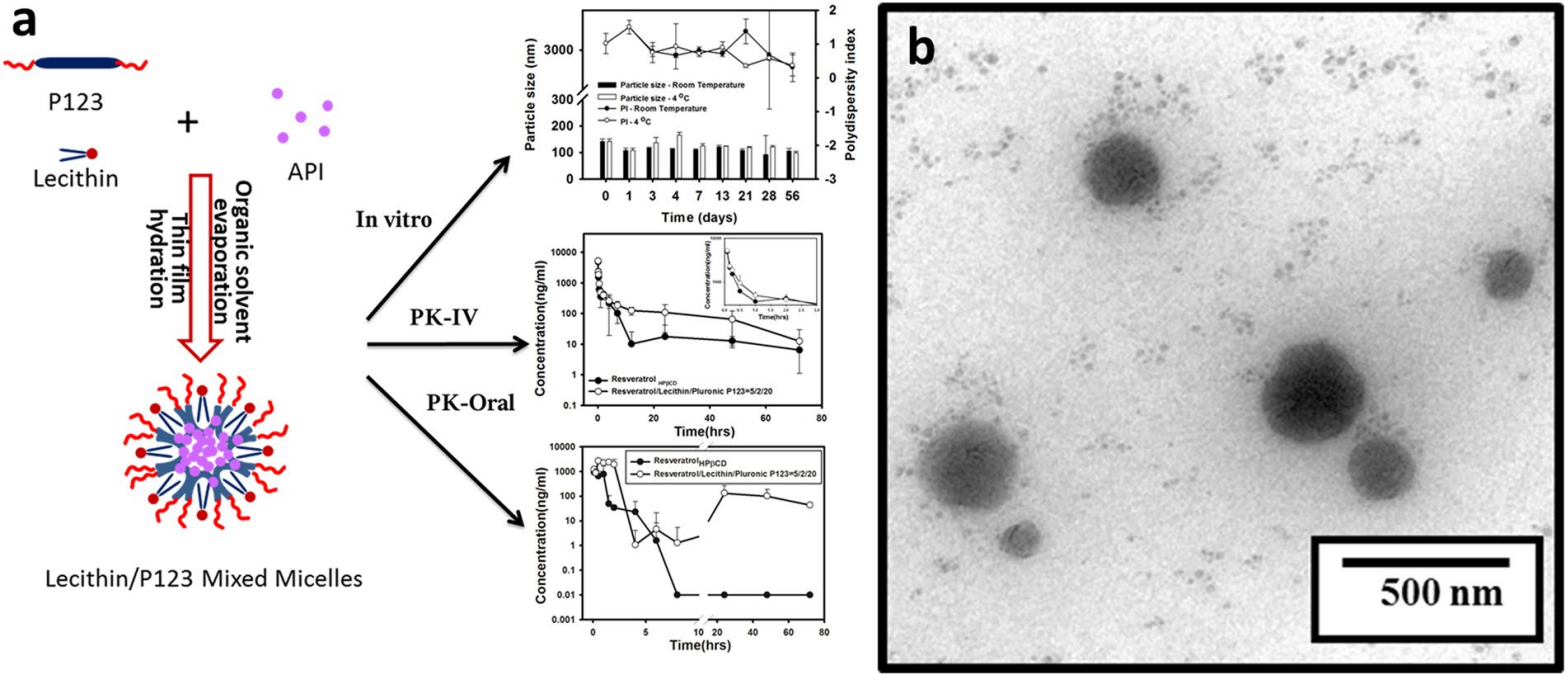
The idea of *sa*LMPMs production was schematically summarized and the significant results were highlighted (**a**). TEM micrograph of *trans*-resveratrol (*t*-Rev)-loaded PP123 self-assembling lecithin-based mixed polymeric micelles (*sa*LMPMs) (**b**).

In light of those reports, we wanted to explore the feasibility of moving *t*-Rev into clinical evaluations as a cancer chemopreventive agent through the use of self-assembling lecithin-based mixed polymeric micelles (*sa*LMPMs), which were successfully applied to enhance the solubility and oral bioavailability of quercetin and curcumin^24, 25^. By incorporating extra hydrophobic materials of lecithin with amphiphilic polymers, the volume of the hydrophobic core of LMPMs increases and provides a larger solubilization space for hydrophobic drugs, resulting in improved solubility. Lecithin, a hydrophobic mixture of naturally occurring phospholipids, is widely applied in the food and pharmaceutical industries and is considered a safe and biocompatible excipient. Lecithin, as a kind of phospholipid, functions as a crucial component of cell membranes to maintain membrane fluidity and an absorption enhancer to facilitate drug absorption. Lecithin as a base and mixed polymeric micelles incorporating additional various amphiphilic polymers, namely sodium deoxycholate (NaDOC), d-alpha tocopheryl polyethylene glycol succinate (TPGS), Cremorphor, or a Pluronic series, were examined in this study for potentially self-assembly to form *sa*LMPMs. We attempted to develop *t*-Rev-loaded micelles with a particle size of <200 nm with a preferable encapsulation efficiency (EE) and drug loading (DL). In addition, the physicochemical (morphological observations and *in vitro* drug release) and both oral and intravenous pharmacokinetic (PK) properties of optimal *t*-Rev-loaded *sa*LMPMs were characterized. Figure 1a schematically summarizes the idea of *sa*LMPMs production and highlights the significant results.

## Results

We first screened various amphiphilic polymers to combine with lecithin for formulating *t*-Rev-loaded *sa*LMPMs and eventually determined an optimal formulation to achieve a desirable smaller size and maximal EE and DL.

### Screening and optimization of *t*-Rev-loaded *sa*LMPMs

Particle size is a crucial parameter because it directly affects the physical stability, cellular uptake, biodistribution, and drug release from micelles, resulting in different bioavailabilities and pharmacokinetic profiles with both oral and intravenous administration. The PI is a dimensionless measure of the breadth of the particle size distribution^26, 27^. Table 1 lists preliminary screening results of *t*-Rev-loaded *sa*LMPMs formed with different ratios of various amphiphiles and lecithin. It indicates that with NaDOC and TPGS, a higher ratio of NaDOC (1:0:20) was required to encapsulate *t*-Rev in MPMs with a size of <50 nm without precipitation within 12 h. The addition of lecithin (1:1:20), however, did not improve the EE and contrarily caused precipitation of *t*-Rev. Table 1 further demonstrates that with the Pluronic^®^ Flake series (PF68, PF108, PF87, and PF127), higher ratios of these amphiphiles (1:0:20) was better for the EE of *t*-Rev in LMPMs without precipitation within 12 h. Similarly, the addition of lecithin (1:1:20) was found to enlarge the volume of LMPMs for drug encapsulation resulting in an increase in the micellar size but causing some precipitation within 12 h. Table 1 also shows that PL121 was the only amphiphile that did not form *t*-Rev-loaded LMPMs, even if its ratio was increased or lecithin was added. As illustrated by Table 1, both CRH40 and CELP formed *t*-Rev-loaded *sa*LMPMs. However, the addition of lecithin seemed to increase the micellar size compared to that only containing the amphiphile, indicating that the volume of micelles had expanded. Nevertheless, expansion of the micellar volume did not provide accommodation for loading *t*-Rev as indicated by the fact that precipitation occurred within 12 h for those *t*-Rev-loaded LMPMs.

**Table 1:** Preliminary screening of mixed micelle formation with various ratios of different amphiphiles (NaDOC; TPGS, Pluronic^®^ P123, F68, F87, F127, F108, and L121; Cremophor^®^ RH40, and ELP) and lecithin.

Amphiphiles (HLB)	Resveratrol:Lecithin:Amphiphile					
	1:0:5	1:1:5	1:0:10	1:1:10	1:0:20	1:1:20
NaDOC (16)	>3000	>3000^c^	310.3 ± 57.47^a^ (1.023 ± 0.367)^b^	>3000	110.3 ± 12.61 (1.697 ± 0.096)	1334.8 ± 1054.63 (1.696 ± 0.472)
TPGS (13)	>3000	64.1 ± 1.66 (0.978 ± 0.166)	2586.5 ± 698.41 (0.341 ± 3.802)	>3000	24.6 ± 17.26 (−1.93 ± 3.65)	761.2 ± 1047.52 (1.738 ± 0.528)
F68 (>24)	>3000	636.7 ± 58.47 (1.518 ± 0.031)	>3000	366.1 ± 5.61 (1.07 ± 0.054)	7.3 ± 5.2 (−2.355 ± 5.141)	368.4 ± 10.68 (0.598 ± 0.145)
F108 (>24)	>3000	533.3 ± 4.23 (0.978 ± 0.032)	32.2 ± 12.01 (0.5 ± 0.889)	363.5 ± 6.88 (0.545 ± 0.077)	193.1 ± 190.86 (−0.391 ± 1.628)	391.5 ± 3.29 (0.721 ± 0.05)
F87 (>24)	>3000	226.6 ± 0.83 (0.989 ± 0.033)	10 ± 5.49 (1.055 ± 0.556)	338.6 ± 11.59 (1.218 ± 0.049)	271.1 ± 124.85 (0.403 ± 0.649)	759.5 ± 46.34 (0.277 ± 0.147)
F127 (18–23)	>3000	425 ± 0.45 (1.369 ± 0.026)	612.4 ± 25.13 (0.892 ± 0.05)	340.9 ± 11.27 (1.566 ± 0.015)	22 ± 1.43 (1.308 ± 0.262)	477.7 ± 139 (1.534 ± 0.052)
L121 (1–7)	>3000	>3000	>3000	>3000	>3000	>3000
P123 (7–9)	104.4 ± 2.63 (0.217 ± 0.064)	145.6 ± 1.42 (0.578 ± 0.007)	75.4 ± 2.1 (0.564 ± 0.148)	203.8 ± 76.15 (1.231 ± 0.318)	24.3 ± 0.88 (1.465 ± 0.211)	104.6 ± 4.34 (1.595 ± 0.025)
CRH40 (14.3)	341.1 ± 162.06 (1.242 ± 0.269)	432.2 ± 3.46 (0.8 ± 0.041)	32.1 ± 3.13 (1.232 ± 0.093)	890.1 ± 19.56 (0.946 ± 0.025)	14.9 ± 10.51 (−3.803 ± 6.232)	350.5 ± 15.1 (1.639 ± 0.008)
CELP (13.9)	164.6 ± 1.57 (0.755 ± 0.01)	235 ± 3.24 (0.758 ± 0.029)	20.3 ± 1.03 (−0.862 ± 0.634)	302.5 ± 8.194 (1.298 ± 0.042)	18.1 ± 13.93 (0.826 ± 0.758)	188.4 ± 13.01 (1.916 ± 0.011)

^a^Mean size ± SD; ^b^Mean PI ± SD; ^c^underline stander for precipitation during 12 hr.

As revealed in Table 1, PP123 was able to form *t*-Rev-loaded LMPMs at these three ratios (1:0:5/1:1:5, 1:0:10/1:1:10, and 1:0:20/1:1:20). The EE increased with an increasing ratio of PP123, as indicated by no precipitation occurring within 12 h. The micellar size increased with the addition of lecithin, revealing that the increase in micellar volume allowed a greater amount of *t*-Rev to be encapsulated without precipitation. PP123 is a XYX triblock amphiphilic polymer with an average number of EO units (2X) of 39.20 and an average number of PO units (Y) of 69.40. Its molecular mass is 5750 Da with a hydrophilic-hydrophobic balance (HLB) of 8 and a critical micellar concentration (CMC) of 4.4 µM. This might be explained by such physical characteristics of PP123 making it compatible with lecithin to self-assemble mixed micelles with sufficient volume to load higher amounts of *t*-Rev without precipitation. Therefore, PP123 was selected for further optimization of the self-assembling micellar formulation with a particle size of <200 nm, an EE of >90%, and a DL of >15%.

Table 2 illustrates the physical characteristics, including the mean particle size, PI, DL, and EE, for those corresponding micelles formed utilizing various ratios of *t*-Rev/Lec/PP123. Results clearly indicate that when PP123 was used at the highest amount of 20 mg, an amount of Rev of as high as 5 mg could be loaded into the micelles forming stable *t*-Rev-loaded *sa*LMPMs with nearly a 100% EE and close to 20% DL. At a *t*-Rev/PP123 ratio of 5/20, it was also confirmed that with the addition of lecithin, the micellar volume of micelles increased resulting in slight increases in the EE and DL without precipitation. The optimal formulation ratio for *t*-Rev/Lec/PP123 was found to be 5:2:20. This optimal formulation was designated *t*-Rev-loaded PP123 *sa*LMPMs and was then selected for further evaluation.

**Table 2:** Particle size, polydispersity index (PI), drug loading (DL), and encapsulation efficiency (EE) of *trans*-resveratrol (*t*-Rev)-loaded self-assembling lecithin-based mixed polymeric micelles (*sa*LMPMs) formed using *t*-Rev, lecithin, and PP123 in various ratios.

Rev:L:PP123	Particle size (nm)	P.I.	D.L. (%)	E.E. (%)
1:0:5	104.4 ± 2.63	0.217 ± 0.064	14.12	84.74
1:1:5	145.6 ± 1.42	0.578 ± 0.007	11.81	82.66
1:0:10	75.4 ± 2.1	0.564 ± 0.148	5.38	59.15
1:1:10	203.8 ± 76.15	1.231 ± 0.318	7.45	89.35
3:1:10	129 ± 8.47	0.289 ± 0.119	17.25	80.51
3:2:10	153.6 ± 35.44	0.989 ± 0.31	17.38	86.90
1:0:20	24.3 ± 0.88	1.465 ± 0.211	4.77	100.18
1:1:20	104.6 ± 4.34	1.595 ± 0.025	4.75	104.54
4:1:20	102.8 ± 3.81	0.754 ± 0.046	14.86	92.86
5:1:20	112.6 ± 9.2	0.3 ± 0.24	18.69	97.17
5:2:20	147.7 ± 9.62	0.842 ± 0.076	18.52	103.93

### Physical Characterization

**Figure 2:**
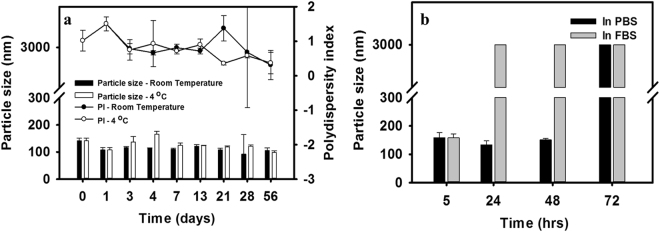
Thermal stability of *trans*-resveratrol (*t*-Rev)-loaded PP123 self-assembling lecithin-based mixed polymeric micelles (*sa*LMPMs) stored at room temperature or 4 °C in the dark (**a**) and serum stability in phosphate-buffered saline and fetal bovine serum at 37 °C (**b**).

TEM as shown by Fig. 1b revealed that the particle size of the optimal formulation (*t*-Rev-loaded PP123 *sa*LMPMs) was around 150 nm with a broader distribution, which was consistent with that measured by DLS which showed a higher PI value of 0.842 ± 0.076. The thermal stability of optimal *t*-Rev-loaded PP123 *sa*LMPMs was determined by subjecting them to storage at room temperature and 4 °C, while stability in serum was evaluated by incubation in PBS and FBS at 37 °C. Changes in the particle size with/without the PI value were examined in the stability test, and results are shown in Fig. 2. As to thermal stability, *t*-Rev-loaded PP123 *sa*LMPMs stored at both temperatures exhibited no obvious changes in the particle size or size distribution for at least 2 months (Fig. 2a). Regarding serum stability at 37 °C, the particle size of *t*-Rev-loaded PP123 *sa*LMPMs incubated with FBS increased to >200 nm at 5 h, while the particle size for *t*-Rev-loaded PP123 LMPMs in PBS did not exceed 200 nm until 24 h (Fig. 2b).

### *In vitro* release studies

**Figure 3:**
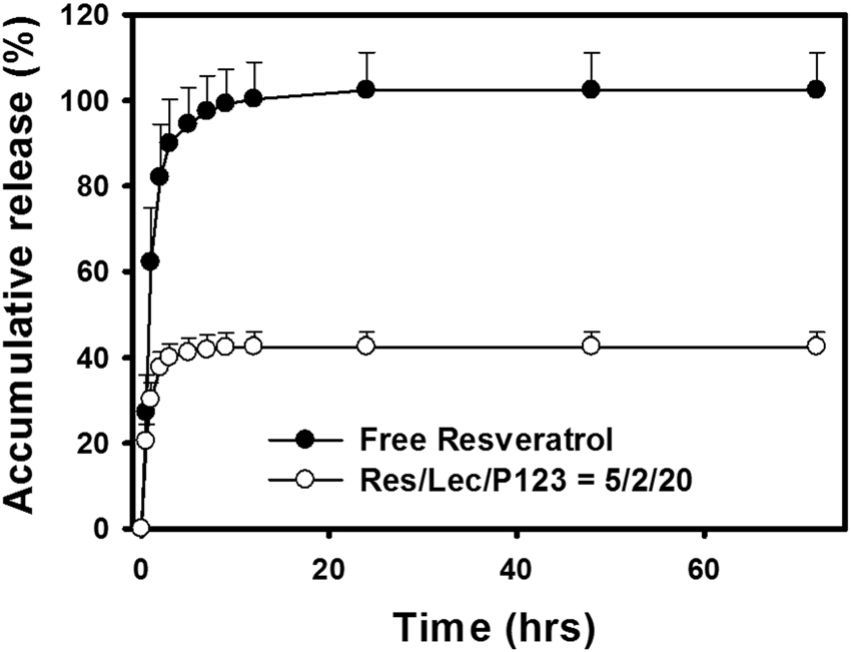
*In vitro* release profiles of *trans*-resveratrol (*t*-Rev) from a free *t*-Rev solution and *t*-Rev-loaded PP123 self-assembling lecithin-based mixed polymeric micelles (*sa*LMPMs) (*n* = 3).

Figure 3 illustrates that the release of *t*-Rev from a free *t*-Rev solution was fast and reached a plateau of complete release within 12 h. The initial release of *t*-Rev from *t*-Rev-loaded PP123 *sa*LMPMs was similar to that released from the free *t*-Rev solution, but only 40% of the total content was released within the same period of time. Since *t*-Rev was completely dissolved in a free *t*-Rev solution, the rapid and complete release of *t*-Rev from the free *t*-Rev solution was expected. However, when *t*-Rev was encapsulated in *t*-Rev-loaded PP123 *sa*LMPMs, its release was hindered, and only those portions induced by micelle dilution and/or loosely adsorbed onto micelles were released.

### *In vivo* PK studies

**Figure 4:**
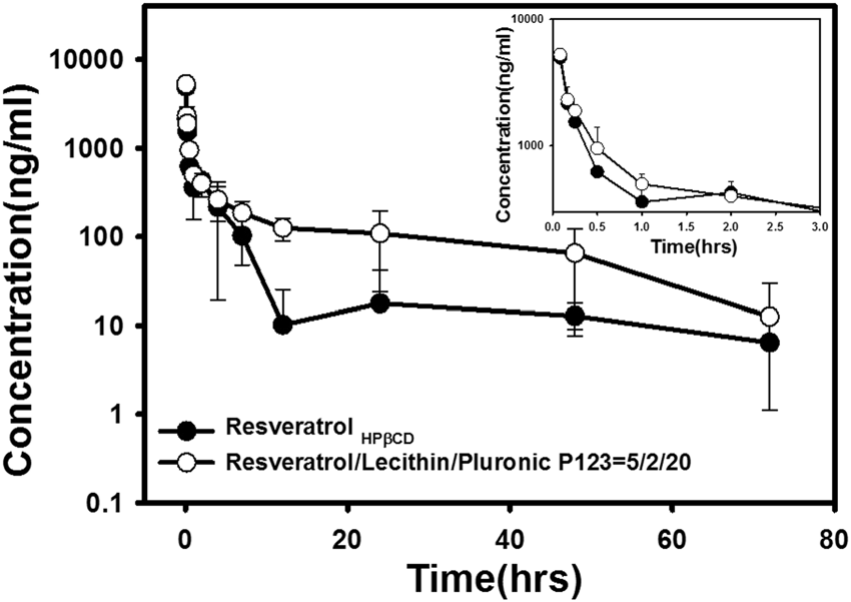
Plasma concentration-time curves of *trans*-resveratrol (*t*-Rev) after intravenous administration of *t*-Rev-loaded PP123 self-assembling lecithin-based mixed polymeric micelles (*sa*LMPMs) and a *t*-Rev/HP-β-CD complex solution (20 mg/kg) to rats. Each point represents the mean ± SD of three determinations (*n* = 3).

Results of *in vivo* PK studies with an IV injection are illustrated in Fig. 4, which shows that after IV administration of the *t*-Rev/HP-β-CD complex solution, the *t*-Rev concentration quickly dropped to 10 ng/mL and then remained at this level for 72 h; however, after IV delivery of *t*-Rev-loaded PP123 *sa*LMPMs, a slower decay of *t*-Rev concentration to 100 ng/mL was observed, which then maintained this level or slightly decreased below it for 72 h. PK parameters for IV administration of the *t*-Rev/HP-β-CD complex solution and *t*-Rev-loaded PP123 *sa*LMPMs are summarized in Table 3. It shows that PP123 *sa*LMPMs reduced the elimination rate constant (*K*_e_) and increased the elimination half-life (*t*_1/2_) of *t*-Rev. The absolute BA increased 2.17-fold for *t*-Rev-loaded PP123 *sa*LMPMs with respect to the *t*-Rev/HP-β-CD complex solution.

**Table 3:** Summary of the pharmacokinetic (PK) parameters of *trans*-resveratrol (*t*-Rev) following intravenous or oral administration of *t*-Rev-loaded PP123 self-assembling lecithin-based mixed polymeric micelles (*sa*LMPMs) and a *t*-Rev/HP-β-CD complex solution (*n* = 3).

Treatment Parameters	IV_HPβCD_	IV_PP123_	Oral_HPβCD_	Oral_PP123_
Dose (mg/kg)	20	20	40	40
k_el_ (1/hr)	0.05 ± 0.02	0.03 ± 0.01	0.16 ± 0.07	0.04 ± 0.01
t_1/2_ (h)	17.17 ± 8.40	24.76 ± 6.63	4.76 ± 1.98	17.87 ± 6.15
T_max_ (h)			0.54 ± 0.65	1.00 ± 0.71
C_max_ (μg/ml)			1.28 ± 0.19	2.82 ± 0.14
AUC_0-72_ (h.μg/ml)	4.47 ± 0.29	9.70 ± 4.04	1.10 ± 0.47	12.61 ± 4.06
V (L/kg)	112.2 ± 56.5	84.14 ± 30.64	297.9 ± 229.5	81.5 ± 1.94
CL (L/h/kg)	4.48 ± 0.29	2.30 ± 0.86	39.88 ± 1.63	3.35 ± 1.08
MRT(hr)	13.20 ± 7.38	24.49 ± 2.49	2.12 ± 1.63	17.84 ± 8.90
F_ab_ (%)	100	217	12	141
F_rel_ (%)			100	1144

**Figure 5:**
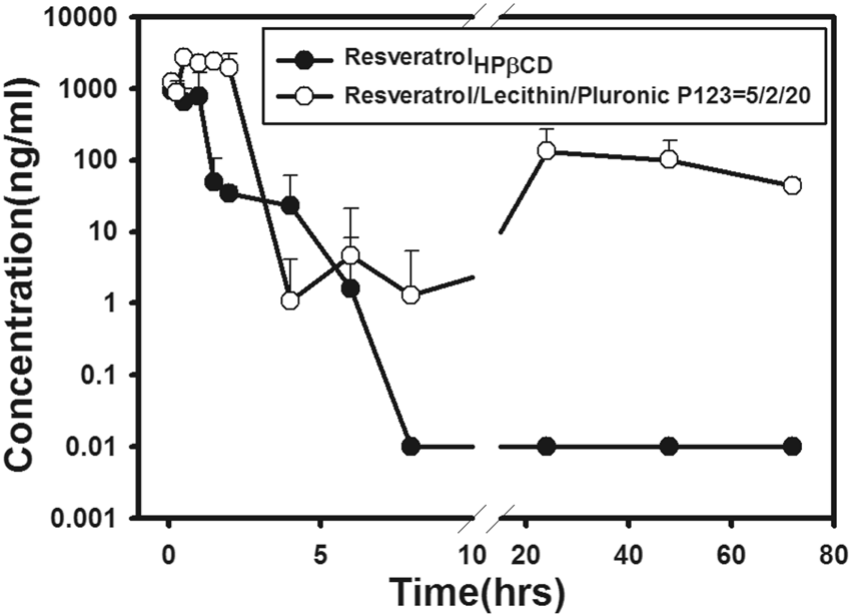
Plasma concentration-time curves of *trans*-resveratrol (*t*-Rev) after oral administration of *t*-Rev-loaded PP123 self-assembling lecithin-based mixed polymeric micelles (*sa*LMPMs) and a *t*-Rev/HP-β-CD solution (50 mg/kg) to rats. Each point represents the mean ± SD of three determinations (*n* = 3).

Results for *in vivo* PK studies of oral administration of the *t*-Rev/HP-β-CD complex solution and *t*-Rev-loaded PP123 *sa*LMPMs are illustrated in Fig. 5, and PK parameters are summarized in Table 3. After oral administration, the maximal concentration of *t*-Rev-loaded PP123 *sa*LMPMs was about 2.2-fold higher than that of the *t*-Rev/HP-β-CD complex solution. Absolute BA values with oral administration were 12% and 141% for the *t*-Rev/HP-β-CD complex solution and *t*-Rev-loaded PP123 *sa*LMPMs, respectively. The relative bioavailability (*F*_rel_) of *t*-Rev-loaded PP123 *sa*LMPMs with respective to *t*-Rev/HP-β-CD complex solution was 11.44-fold higher. In a 3-h time period, the plasma concentration profile after oral administration of *t*-Rev-loaded PP123 *sa*LMPMs maintained a plasma concentration above a minimal concentration of 1150 ng/mL (5 µM) for chemoprevention, whereas no concentrations reached this minimal level after oral administration of the *t*-Rev/HP-β-CD complex solution.

## Discussion

In this study, in order to increase the solubility of *t*-Rev to improve the PK profile and enhance the bioavailability, we utilized *sa*LMPMs composed of lecithin and amphiphilic polymers to encapsulate *t*-Rev. In a preliminarily screening test, it was found that PP123 was the optimal amphiphilic polymer to combine with lecithin. This was attributed to the physical characteristics of PP123 with an HLB of 8 and a CMC of 4.4 µM, making it more compatible with lecithin to facilitate the self-assembly of forming mixed micelles with a sufficient core volume to load higher amounts of *t*-Rev without precipitation. Further optimization of the self-assembling micellar formulation, *t*-Rev:Lec:PP123, at a 5:2:20 ratio designated *t*-Rev-loaded PP123 *sa*LMPMs, was determined to be optimal with desirable physical characteristics, including a mean particle size of <200 nm, an EE of >90%, and a DL of >15%. Compared to *t*-Rev solubilized with HP-β-CD complexing, *t*-Rev-loaded PP123 *sa*LMPMs efficiently improved *t*-Rev stability in PBS at room temperature, 4 °C, and 37 °C and in FBS at 37 °C, and retarded the *in vitro t*-Rev release. Therefore, this study reports a simple and cost-effective formulation composed of lecithin and PP123 for encapsulating *t*-Rev that is potentially able to achieve therapeutic effectiveness by improving *t*-Rev’s PK profile and bioavailability.

*t*-Rev is a polyphenolic compound and has garnered great interest in pharmaceutical research areas due to its beneficial properties, namely antioxidant, antiradical, anti- inflammatory, anticarcinogenic, antibacterial, and antiviral effects^28, 29^. This broad variety of therapeutic activities, in conjunction with its safety profile (generally recognized as safe status) and natural origin (commonly found in fruits and vegetables), makes *t*-Rev a very attractive candidate for developing novel pharmaceutical products^30^. However, the use of *t*-Rev is limited by its very low bioavailability in humans and animals resulting from its poor water solubility (~50 µg/ml) and enterohepatic recirculation and extensive first-pass metabolism by CYP3A4 in the liver, even though *t*-Rev exhibits higher membrane permeability (a log *P* of 3.1 being considered a class-II compound in the Biopharmaceutical Classification System) with high absorption from the gastrointestinal tract^21^. Therefore, drug supplements containing a high dose of *t*-Rev were developed for oral administration in order to elevate plasma concentrations to therapeutic levels in an attempt to provide its beneficial effects to human health. However, a recent human clinical study to assess the PK properties and safety of *t*-Rev following a single oral dose of 500 mg (one Evelor 500 mg tablet; Agetis Supplements, Limassol, Cyprus) reported that the C_max_ and AUC_0-inf_ were lower for *t*-Rev with respective values of 71.2 ± 42.4 ng/ml and 179.1 ± 79.1 ng/ml/h, but no adverse reactions associated with *t*-Rev were observed during the study^1^. The plasma concentration profile of *t*-Rev clearly showed that all plasma concentrations after oral administration of a higher dose of 500 mg fell far short of achieving a therapeutic level of 5 µM for chemoprevention.

To resolve the low oral bioavailability of *t*-Rev leading to less-desirable plasma concentrations, IV administration of *t*-Rev is an alternative of choice. Being solubilized by complexing with HP-β-CD, Marier *et al*. first reported that after IV administration of 15 mg/kg of the *t*-Rev/HP-β-CD complex, plasma concentrations of *t*-Rev declined rapidly over the first hour with an elimination half-life (T_1/2_) of 0.13 h, which was followed by the appearance of a second peak at 2~4 h and then a slow decline in the plateau phase after administration^31^. This phenomenon is well-documented^8, 19, 22, 32–34^. Based on a study reported by Colom *et al*., a fast decline in the plasma profile of *t*-Rev within the first hour after IV administration with a short half-life value was attributed to its high clearance and large distribution volume values. The reappearance of a second peak after the initial decline of *t*-Rev was confirmed to be enterohepatic recirculation, and the slow decline in the plateau phase might be due to steady-state equilibrium between the peripheral compartment with a larger volume value (2.80 L) and the central compartment with a smaller volume vale (0.249 L)^32^.

The plasma profile with a plateau concentration of about 10 ng/ml observed for IV administration of 20 mg/kg *t*-Rev solubilized with the HP-β-CD complex as shown in Fig. 5 in this study conformed to that reported by Das *et al*. Those studies of IV administration of the *t*-Rev/HP-β-CD complex indicated that a therapeutic *t*-Rev level of 5 µM for chemoprevention could only be maintained for a very short period of time (less than 15 min) after administration due to the rapid decline over the first hour as a result of *t*-Rev being largely metabolized to two metabolites, glucuronide and sulfate, and being extensively distributed to the peripheral compartment. Nevertheless, the plasma profile with IV administration of 20 mg/kg *t*-Rev-loaded PP123 *sa*LMPMs as shown in Fig. 5 displayed a similar plasma profile but with a higher plateau concentration of about 100 ng/ml. A slightly extended time period with the plasma concentration of *t*-Rev above the therapeutic level for chemoprevention was observed with IV administration of 20 mg/kg *t*-Rev-loaded PP123 *sa*LMPMs, which was likely due to the same reason as that for IV administration of the *t*-Rev/HP-β-CD complex. However, the result of a 10-fold higher plateau concentration might indicate that following IV administration of *t*-Rev-loaded PP123 *sa*LMPMs, the steady-state plateau concentration of *t*-Rev in the peripheral compartment would be higher, implying that a greater portion of *t*-Rev was preferably distributed to the target site located in the peripheral compartment. Several studies that utilized various nanocarriers, e.g., liposomes, nanoparticles, and nanoemulsions, illustrated a similar plateau pattern of plasma profiles after IV administration of *t*-Rev-loaded nanocarriers as that for *t*-Rev-loaded PP123 *sa*LMPMs^21, 35, 36^. Therefore, it was concluded that although IV administration of *t*-Rev either solubilized with HP-β-CD or encapsulated in *sa*LMPMs was inherently unable to establish a therapeutic level for chemoprevention in a reasonable time period to achieve health benefits, *t*-Rev-loaded PP123 *sa*LMPMs were able to preferably distribute a greater portion of *t*-Rev to the peripheral compartment, potentially establishing a therapeutic level at the targeted site.

As to translating *t*-Rev into practical usefulness, delivery by the oral route would be the best choice. Oral formulations of *t*-Rev solubilized with either HP-β-CD derivatives (HP-β-CD and RM-CD), cosolvents (DMSO, PEG300, and PEG400), or surfactants (Tween 80) demonstrated enhanced *t*-Rev oral bioavailability (expressed as the dose (D, mg/kg)-normalized AUC (µM·h): AUC/D = 0.08~0.40) over the traditional suspension form of pure *t*-Rev in male Wistar rats, but mostly only furnishing *t*-Rev levels in plasma at concentrations below or considerably below 5 µM (~1150 ng/mL) required to elicit pharmacologic effects relevant to chemoprevention^8, 11, 15, 16, 19–22, 31–33, 37^. The plasma concentration profile after oral administration of *t*-Rev/HP-β-CD in male SD rats in this study was found to conform to that revealed above with an AUC/D value of around 0.12 (as shown in Table 3). This indicates that the PK study performed herein was consistent with that reported in the literature.

Among *t*-Rev-loaded nanocarrier systems, including solid lipid nanoparticles^18, 19^, polymeric nanoparticles^20–22^, nanoemulsions^11, 16^, and solid dispersions in dripping pills^15^ developed for oral administration, only those nanocarrier systems reported by Pandita *et al*.^18^, Penalva *et al*.^22^, and Zhou *et al*.^16^ showed enhanced bioavailability with either a longer period of time with the plasma concentration above a desirable level for chemoprevention, or an AUC/D value much greater than 1.0, or both. Compared those reported nanocarrier systems for oral administration, *t*-Rev-loaded PP123 *sa*LMPMs revealed in this study furnished a 3-h period of time when the plasma concentration of *t*-Rev remained above the desirable plasma concentration for chemoprevention and an AUC/D value of 1.38, which established a 40% increase in the absolute bioavailability (with respect to IV administration of the *t*-Rev/HP-β-CD complex) and a 11.4-fold increase of the relative bioavailability (with respect to the oral administration of the *t*-Rev/HP-β-CD complex). With these PK characteristics, it was expected that both intravenous and oral administration of *t*-Rev-loaded PP123 *sa*LMPMs at a reasonable dose will be able to achieve health benefits in humans.

## Conclusion

Considering the potential of *t*-Rev to provide health benefits, the present study involved developing and characterizing *t*-Rev-loaded *sa*LMPMs to improve *t*-Rev’s PK profiles and bioavailability. The optimal formulation of *t*-Rev-loaded PP123 *sa*LMPMs was composed of *t*-Rev:lecithin:PP123 at a 5:2:20 ratio and provided a particle size of <200 nm, an EE of >90%, and a DL of >15%. IV administration of *t*-Rev-loaded PP123 *sa*LMPMs revealed that a greater portion of *t*-Rev was preferably distributed into the peripheral compartment, potentially establishing a therapeutic level at the targeted site. For oral administration, *t*-Rev-loaded PP123 *sa*LMPMs furnished a 3-h period of time when the plasma concentration of *t*-Rev remained above the desired plasma concentration for chemoprevention and potentially established a therapeutically effective level at the target site. It was concluded that *sa*LMPMs are a simple and cost-effective formulation for encapsulating *t*-Rev that is potentially able to achieve therapeutic effectiveness by improving *t*-Rev’s PK profile and bioavailability. Ultimately, the process of producing lecithin-based *sa*LMPMs is also simple and easily reproducible and can be scaled-up to make the process operable on a large scale.

## Materials and Methods

### Materials

*t*-Rev (3,4′,5-trihydroxy-*trans*-stilbene), sodium deoxycholate (NaDOC), Pluronic^®^ L121 (PL121), F108 (PF108), and P123 (PP123) were purchased from Sigma-Aldrich (St. Louis, MO, USA). Lecithin (Lipoid S-100) were supplied by Lipoid AG (Steinhausen/ZG, Switzerland). Pluronic^®^ F87 (PF87), F127 (PF127), and F68 (PF68); TPGS; and Cremophor^®^ ELP (CELP) and RH40 (CRH40) were purchased from BASF (Hanover, Germany). DSPE-PEG2K was obtained from NOF (Tokyo, Japan), and heparin (5000 IU/mL) was provided by China Chemical & Pharmaceutical (Hsinchu, Taiwan). Hydroxypropyl-β-cyclodextran (HP-β-CD, with a degree of substitution of ∼0.6) was supplied by Roquette Freres S.A. (Lestrem, France). All reagents for the high-performance liquid chromatography (HPLC) and ultra-performance liquid chromatography (UPLC)/tandem mass-spectrometry (MS/MS) analyses were of an HPLC or MS grade, and other reagents were of an analytical grade.

### Preparation of *t*-Rev-loaded *sa*LMPMs

*t*-Rev-loaded *sa*LMPMs were prepared using a thin film method as previously reported^24, 25^. Briefly, 1 mL of a mixed solvent (methanol: dichloromethane, 3:7, v/v) in a 10-ml flask was used to dissolve *t*-Rev, lecithin, and one of the amphiphilic polymers (NaDOC, PF87, PF127, PF68, PL121, PF108, PP123, TPGS, CRH40, or CELP) at a predetermined ratio. The mixture was shaken for 30 s, sonicated for 1 min, and subsequently evaporated through rotary evaporation (Buchi, Rotavapor R124, Hamilton Instrument, Cinnaminson, NJ, USA) under reduced pressure to remove the solvent and obtain a thin film. After adding 1 mL of deionized water, the thin film was gently shaken until completely dispersed to induce the self-assembly of micelles. Unincorporated *t*-Rev aggregates were removed by passing the solution through a 0.22-µm filter (Millipore, Billerica, MA, USA). Characteristics of the *t*-Rev-loaded *sa*LMPMs, namely the average particle size and size distribution (polydispersity index, PI), EE, and DL, were determined.

### Characterization of *t*-Rev-loaded *sa*LMPMs

The average particle size and size distribution of *t*-Rev-loaded *sa*LMPMs were measured using an N5 submicron particle size analyzer (Beckman Coulter, Brea, CA, USA) at room temperature. The surface morphology was observed through transmission electron microscopy (TEM; Hitachi H-600, Tokyo, Japan).

### Quantification of *t*-Rev

*t*-Rev was analyzed using an HPLC method (Pump PU-980, Jasco, Tokyo, Japan) adapted from Chen *et al*. with slightly modifications^38^. The *t*-Rev concentration was determined using an XBridge^TM^ C18 column (5 μm, 150 × 4.6 mm, Waters, Milford, MA, USA). The mobile phase was a mixture of methanol and 0.5% acetic acid (4:6, v/v) at a flow rate of 1.0 mL/min at 30 °C. Furthermore, the column effluent was monitored using an ultraviolet detector (UV-975, Jasco) at a wavelength of 303 nm, and the HPLC method was validated to have acceptable intraday (coefficient of variation (CV): 1.56%~3.51%, relative standard error (RSE): −1.34%~1.92%) and interday (CV: 3.39~4.19%, RSE: −1.00~4.41%) CVs for accuracy and precision. After determining the *t*-Rev concentration from the validated calibration curve in the linear range of 0.36~12 μg/mL, the EE and DL were respectively calculated according to equations (1) and (2):

1 $${\rm{Encapsulation}}\,{\rm{efficiency}}\,( \% ,{\rm{EE}})={{\rm{W}}}_{{\rm{M}}}/{{\rm{W}}}_{{\rm{I}}}\times 100\,{\rm{and}}$$

2 $${\rm{Drug}}\,{\rm{loading}}\,( \% ,{\rm{DL}})={{\rm{W}}}_{{\rm{M}}}/({{\rm{W}}}_{{\rm{P}}}+{{\rm{W}}}_{{\rm{M}}})\times 100;$$

where W_M_ is the drug weight in micelles, W_I_ is the weight of the initial feeding drug, and W_P_ is the weight of the initial feeding polymers.

### Stability test

*t*-Rev-loaded *sa*LMPMs were stored at room temperature or 4 °C in the dark. At a predetermined time point, the particle size of the *t*-Rev-loaded *sa*LMPMs was analyzed to evaluate the stability of the product. Equal volumes of *t*-Rev-loaded *sa*LMPMs and phosphate-buffered saline (PBS; 0.01 M, pH 7.4) or fetal bovine serum (FBS) were co-incubated in a 37 °C water bath. At a predetermined time point, the particle size of the *t*-Rev-loaded *sa*LMPMs was analyzed to evaluate their stability in plasma.

### *In vitro* release studies

Drug release from the *t*-Rev-loaded *sa*LMPMs was assessed using the dialysis bag method, in which 0.01 M PBS containing 0.5% Tween 80 was used. One milliliter of *t*-Rev-loaded *sa*LMPMs or a free *t*-Rev solution (i.e., *t*-Rev dissolved in dimethyl sulfoxide, DMSO) diluted with water to a final concentration of 0.1 mg/mL was placed in a separate dialysis bag (MWCO 3500; Cellu-Sep^®^ T1, Membrane Filtration Products, Inc., Seguin, Texas, USA). The bag was placed in a tube, 20 mL of a dissolution medium was added, and the bag was placed at 37 °C at a shaking rate of 100 rpm. At 0.5, 1, 2, 3, 5, 7, 9, 12, 24, 48, and 72 h, the concentration of *t*-Rev released from the dialysis bag was analyzed using the HPLC method, as described in Section 2.4. All measurements were conducted in triplicate. For comparison, *t*-Rev released from a free solution under the same conditions was assessed.

### *In vivo* PK studies

This study involved an animal experiment that was approved by the Institutional Animal Care and Use Committee of Taipei Medical University (approval no.: LAC-2015-0232) and conducted in compliance with Taiwan’s *Animal Welfare Act*. We used 8~10-week-old male Sprague-Dawley (SD) rats to investigate the PK profile of the optimal *t*-Rev-loaded *sa*LMPM formulation (6 mg/mL) and a *t*-Rev/HP-β-CD complex solution (i.e., 60 mg *t*-Rev solubilized in 100 mM of a HP-β-CD complex solution to yield a final concentration of 6 mg/mL). A single intravenous dose of 20 mg/kg of *t*-Rev *sa*LMPMs or the *t*-Rev/HP-β-CD complex solution was given to male SD rats (*n* = 3/group). Blood samples were collected in heparinized tubes from the jugular vein at 0.083, 0.166, 0.25, 0.5, 1, 2, 4, 7, 12, 24, 48, and 72 h after administration. In addition, either a single dose of 40 mg/kg of *t*-Rev-loaded *sa*LMPMs or the *t*-Rev/HP-β-CD complex solution was orally delivered to male SD rats (*n* = 3/group). Blood samples were collected in heparinized tubes from the jugular vein at 0.0833, 0.25, 0.5, 1, 1.5, 2, 4, 6, 8, 12, 24, 48, and 72 h after administration. All blood samples were immediately centrifuged at 3000 rpm for 15 min at 4 °C to obtain plasma, which was stored at −80 °C before the UPLC/MS/MS analysis.

The UPLC/MS/MS analysis was performed according to a method reported by Chen *et al*.^38^ utilizing the Waters ACQUITY UPLC and Xevo TQ MS system equipped with an electrospray ionization (ESI) source. With an injection volume of 20 μL, the HPLC system equipped with a BEH C_18_ column (2.1 mm I.D. × 50 mm, 1.7 μm; Waters) delivered the mobile phase comprised of 25% acetic acid (0.5%) and 75% methanol at a constant flow of 0.25 mL/min to analyze the *t*-Rev concentration. During analyses, ESI parameters were set as follows: a capillary voltage of 3.6 kV for the negative mode; a desolvation temperature of 350 °C; cone gas flow of 100 L/h; and desolvation gas flow of 650 L/h. The UPLC/MS/MS method was validated to have an acceptable of intraday (coefficient of variation (CV): 1.09%~7.71%, relative standard error (RSE): −1.69%~14.25%) and interday (CV: 4.92%~13.08%, RSE: −1.33%~13.21%) CVs for accuracy and precision in the linear concentration range of 10~1000 ng/mL.

PK parameters were estimated through a noncompartmental analysis and are reported as the mean ± standard deviation (SD) from individual rats in each group. The terminal elimination rate constant (*K*_e_) was estimated from the slope of the log-linear phase of a graph of the declining plasma concentration of *t*-Rev versus time. The half-life (T_1/2_) was calculated using the following equation: T_1/2_ = ln 2/*K*_e_. Furthermore, the area under the receiver operator characteristic curve (AUC) from time zero to the last sampling time point (AUC_0→last_) was calculated using the trapezoidal method. Summing AUC_0→last_ and the concentration at the last measured point divided by *K*_e_ yielded AUC_0→∞_. Clearance (CL) was calculated by dividing the dose by AUC_0→∞_, and the distribution volume (V) was calculated by dividing CL by *K*_e_.

### Statistical analysis

Data are presented as the mean ± SD. Student’s *t*-test was used to assess unequal variances. A 2-tailed *p* value of <0.05 was considered statistically significant.
